# Beyond anti-VEGF: expanding the therapeutic horizon and biomarker landscape in retinopathy of prematurity

**DOI:** 10.3389/fped.2025.1713783

**Published:** 2026-01-30

**Authors:** Yuxin Zhang, Qian Chen, Jianli Lv

**Affiliations:** 1 Department of Neonatology, Shandong Provincial Maternal and Child Health Care Hospital Affiliated to Qingdao University, Jinan, China; 2 Department of Pediatric Cardiology, Shandong Provincial Hospital Affiliated to Shandong First Medical University, Jinan, Shandong, China

**Keywords:** anti-VEGF alternatives, biomarkers, combination therapy, IGF-1, oxidative stress, personalized medicine, retinopathy of prematurity

## Abstract

**Background:**

Retinopathy of prematurity (ROP) remains a leading cause of childhood blindness worldwide. While anti-VEGF therapy has revolutionized ROP treatment, concerns regarding systemic absorption, potential neurodevelopmental impacts, and late reactivation have spurred the quest for alternative therapeutic approaches. This mini-review examines emerging therapeutic targets and novel biomarkers that may transform ROP management beyond the anti-VEGF era.

**Methods:**

We conducted a comprehensive literature review of studies published between 2001 and 2025, focusing on novel therapeutic mechanisms, biomarker discovery, and translational research in ROP.

**Results:**

Several promising therapeutic targets have emerged, including: (1) IGF-1 supplementation and IGF-1/IGFBP3 complex therapy; (2) Omega-3 polyunsaturated fatty acids, demonstrating anti-inflammatory and anti-angiogenic properties; (3) Antioxidant therapies targeting oxidative stress pathways; (4) HIF-stabilizing agents that promote physiological vascularization; (5) Cell-based therapies using mesenchymal stem cells and (6) Non-selective beta-adrenergic receptor blockers (propranolol), which target the sympathetic drive of pathological neovascularization. Novel biomarkers under investigation and advanced imaging biomarkers using OCT angiography. Combination approaches integrating multiple pathways show particular promise.

**Conclusions:**

Emerging therapies targeting multiple pathogenic mechanisms, combined with novel biomarkers for risk stratification and treatment monitoring, show potential for more personalized and effective ROP management. Future research should focus on validating these biomarkers in diverse populations and optimizing combination therapy protocols to minimize treatment burden while maximizing visual outcomes and systemic safety.

## 1 Introduction

Retinopathy of prematurity (ROP) represents a significant global health challenge, affecting approximately 20,000 infants annually and remaining the leading cause of preventable childhood blindness in both developed and developing nations (1, 2). The pathophysiology of ROP involves a biphasic process: an initial phase of vessel growth cessation and vaso-obliteration triggered by relative hyperoxia, followed by a proliferative phase characterized by pathological neovascularization driven by hypoxia-induced factors, particularly vascular endothelial growth factor (VEGF) (3, 4).

The introduction of intravitreal anti-VEGF therapy has fundamentally transformed the treatment landscape for severe ROP. Bevacizumab, ranibizumab, and more recently aflibercept have demonstrated remarkable efficacy in inducing disease regression, often with a single injection (5, 6). The BEAT-ROP study established anti-VEGF therapy as a viable alternative to laser photocoagulation, particularly for zone I disease (7). However, accumulating evidence has raised important concerns about the systemic effects of anti-VEGF agents in premature infants.

Several critical limitations of anti-VEGF monotherapy have emerged. First, systemic absorption of intravitreal anti-VEGF agents leads to suppression of circulating VEGF levels for weeks to months, potentially affecting normal organ development during a critical period (8, 9). Recent studies have reported associations between anti-VEGF treatment and adverse neurodevelopmental outcomes, including lower cognitive scores and increased risk of severe neurodevelopmental impairment at 2 years corrected age (10, 11). Second, late reactivation of ROP following anti-VEGF therapy occurs in 8.3%–18% of cases, necessitating prolonged follow-up and potential retreatment (12, 13). Third, anti-VEGF therapy may delay physiological retinal vascularization, leading to persistent avascular peripheral retina (14).

These concerns have catalyzed intensive research into alternative therapeutic approaches that address ROP pathogenesis through non-VEGF dependent mechanisms. The complex pathophysiology of ROP involves multiple interconnected pathways beyond VEGF, including insulin-like growth factor-1 (IGF-1) deficiency, oxidative stress, inflammation, lipid metabolism dysfunction, and mitochondrial dysfunction (15, 16). This multifactorial nature suggests that targeting alternative or complementary pathways may offer therapeutic advantages while potentially avoiding the systemic risks associated with VEGF suppression.

Furthermore, the identification of reliable biomarkers for ROP risk stratification and treatment response monitoring has become a research priority. Current screening protocols rely primarily on clinical risk factors (gestational age, birth weight) and retinal examination findings, which have limited predictive value for individual patients (17). Novel biomarkers derived from serum proteomics, metabolomics, and molecular imaging techniques promise to enable more personalized approaches to ROP management (18, 19).

This review aims to comprehensively examine the emerging therapeutic landscape in ROP beyond anti-VEGF therapy. While many novel pathways are under investigation, significant research gaps remain, particularly in the large-scale clinical validation of these non-VEGF targets and the standardization of new biomarkers. We synthesize current evidence on novel therapeutic targets, evaluate promising biomarkers for disease prediction and monitoring, and discuss combination therapy approaches that may optimize outcomes while minimizing systemic risks. By highlighting these advances and their current evidentiary limitations, we aim to provide clinicians and researchers with a critical framework for understanding the future directions of ROP management in the era of precision medicine.

## 2 Methods

We conducted a comprehensive literature search using PubMed, MEDLINE, Cochrane Library, and ClinicalTrials.gov databases for articles published between 2001 and 2025. The final database search was performed on January 15, 2025. Search terms included combinations of “retinopathy of prematurity,” “ROP,” “anti-VEGF alternatives,” “novel therapy,” “biomarkers,” “IGF-1,” “omega-3,” “antioxidants,” “stem cells,” “combination therapy,” and “personalized medicine.” We prioritized peer-reviewed original research articles, systematic reviews, meta-analyses, and registered clinical trials investigating non-VEGF therapeutic approaches or novel biomarkers for ROP.

To select relevant studies from the search results, the following criteria were applied. Inclusion criteria encompassed: (1) studies investigating therapeutic mechanisms beyond VEGF inhibition; (2) biomarker discovery or validation studies; (3) clinical trials of novel therapeutic agents; and (4) preclinical studies with clear translational potential. We excluded case reports, conference abstracts without full-text availability, and studies focusing exclusively on anti-VEGF monotherapy. Two independent reviewers screened titles and abstracts, with discrepancies resolved through consensus discussion.

Data extraction focused on therapeutic mechanisms, clinical efficacy, safety profiles, biomarker performance characteristics, and stage of clinical development. For biomarker studies, we assessed sensitivity, specificity, and predictive values when available.

### 2.1 Evidence synthesis and limitations

Given the mini-review format, a formal systematic review or meta-analysis adhering to PRISMA guidelines was not performed. The primary objective was a narrative synthesis of emerging trends rather than an exhaustive quantitative analysis. Consequently, a formal quality assessment or risk of bias score (e.g., Cochrane risk-of-bias tool, QUADAS-2 for biomarkers) was not applied to the included studies. The evidence synthesis instead prioritizes describing the mechanism of action and current development status (e.g., preclinical, pilot study, Phase II RCT). This review acknowledges that many cited studies are preclinical or small-scale pilot trials, and these limitations on evidence strength are discussed within the relevant results sections.

## 3 Results

### 3.1 Literature search results

**Figure 1:**
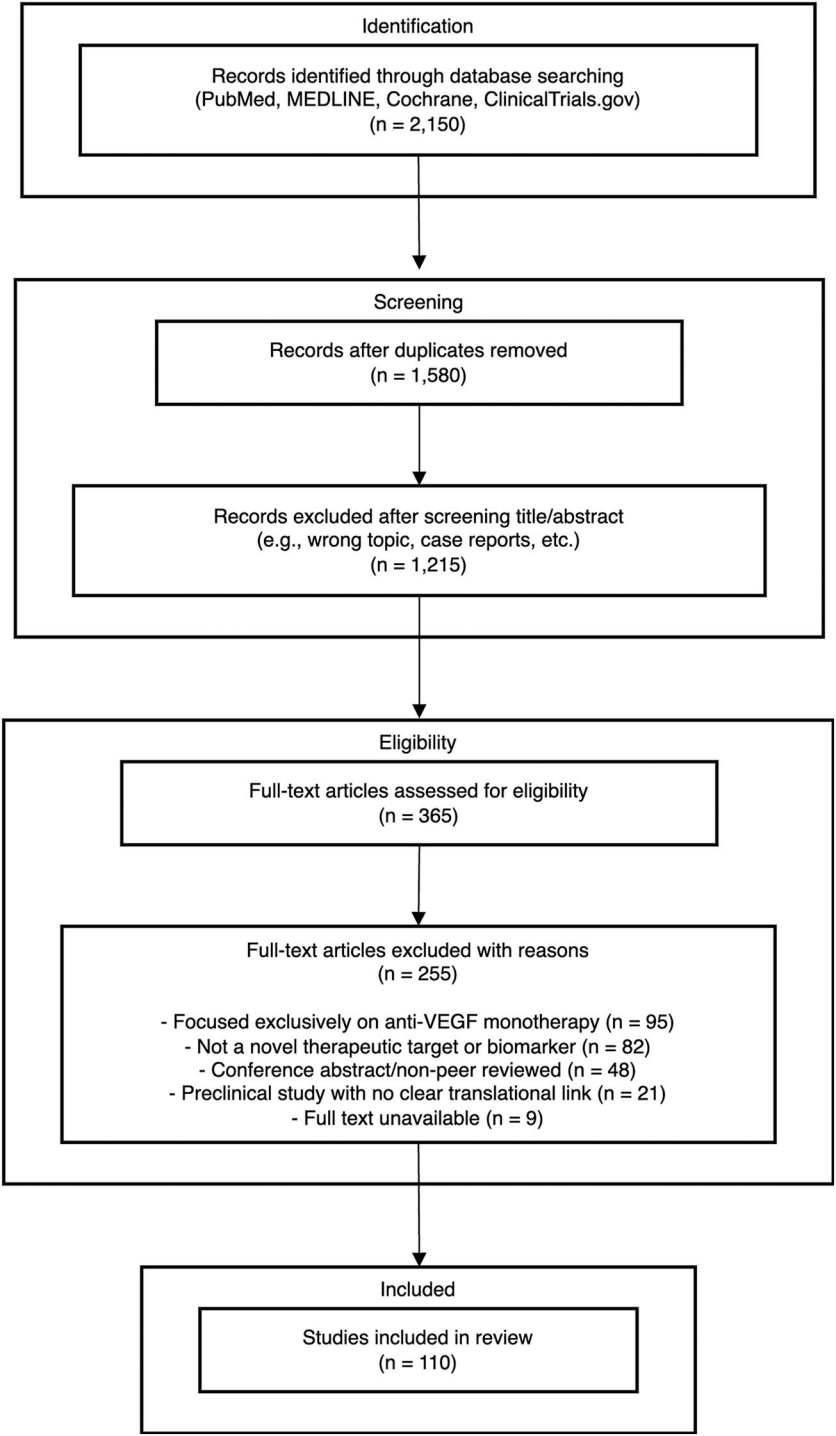
Flow diagram of literature search and selection process.

The literature search process and results are summarized in Figure 1. Initial database searches yielded 2150 potentially relevant citations. After removing duplicates (*n* = 570), 1,580 articles were screened based on titles and abstracts. Of these, 850 were excluded for not meeting inclusion criteria. The remaining 365 articles underwent full-text review, resulting in 255 articles being excluded for various reasons (focused exclusively on anti-VEGF monotherapy, not a novel therapeutic target or biomarker, preclinical study with no clear translational link, or being case reports with limited generalizability). Ultimately, 110 articles were included in this comprehensive review.

### 3.2 Emerging therapeutic targets

#### 3.2.1 IGF-1 and the IGF-1/IGFBP3 complex

Insulin-like growth factor-1 (IGF-1) deficiency represents a fundamental pathogenic mechanism in ROP development. Premature birth interrupts the transplacental transfer of IGF-1, resulting in serum levels lower than *in utero* values (20). This deficiency impairs normal retinal vascular development during the first phase of ROP and exacerbates pathological neovascularization in the second phase (21).

The NIRTURE trial investigated rhIGF-1/rhIGFBP3 complex supplementation in extremely preterm infants. Phase II results demonstrated a 53% reduction in severe bronchopulmonary dysplasia and showed promising trends toward reduced severe ROP incidence (22). Importantly, the treatment appeared safe with no increase in adverse events. Mechanistically, IGF-1 supplementation promotes physiological vascularization through Akt pathway activation while suppressing pathological angiogenesis by modulating VEGF sensitivity (23).

Current research focuses on optimizing IGF-1 delivery and dosing strategies. Continuous intravenous infusion maintains more stable levels compared to subcutaneous administration, potentially improving efficacy (24). Additionally, combining IGF-1 supplementation with nutritional interventions that naturally boost IGF-1 levels, such as enhanced protein intake and breast milk fortification, represents a promising integrated approach (25).

#### 3.2.2 Omega-3 polyunsaturated fatty acids

Long-chain polyunsaturated fatty acids (LC-PUFAs), particularly docosahexaenoic acid (DHA) and eicosapentaenoic acid (EPA), exert multiple protective effects against ROP development. These include anti-inflammatory actions through specialized pro-resolving mediators (SPMs), direct anti-angiogenic effects, and promotion of neuronal and vascular maturation (26). Adequate levels of both Omega-3 and Omega-6 LCPUFA are crucial in protecting against retinal neurovascular dysfunction in a Phase I ROP model; however, supplementation should focus on optimizing the ratio while ensuring adequate Omega-6 levels are maintained. Activation of the APN pathway may further enhance the Omega-3 and Omega-6 LCPUFA's protection against ROP (27).

Recent mechanistic studies have elucidated how omega-3 fatty acids generate specialized pro-resolving mediators including resolvins, protectins, and maresins. These bioactive lipid mediators actively resolve inflammation and promote tissue repair without immunosuppression (28). In oxygen-induced retinopathy models, resolvin D1 and neuroprotectin D1 significantly reduced pathological neovascularization while promoting revascularization of avascular areas (29).

Parenteral lipid emulsions containing fish oil (SMOFlipid) vs. traditional soybean oil-based formulations have shown promise in reducing ROP incidence. A systematic review found that fish oil-containing emulsions significantly reduced severe ROP compared with conventional lipids (30). Current research investigates optimal omega-3:omega-6 ratios and the role of specialized pro-resolving mediator supplementation.

#### 3.2.3 Antioxidant therapies

Oxidative stress plays a central role in ROP pathogenesis, as preterm infants have immature antioxidant systems and experience relative hyperoxia after birth. Multiple antioxidant strategies are under investigation:

N-acetylcysteine (NAC): Preclinical studies in animal models suggest that acetylcysteine can significantly reduce the formation of retinal neovascularization by continuously interfering with the nitration of tyrosine, and can also mediate its antioxidant anti-angiogenic effect by reducing the expression of insulin-like growth factor 1 at the RNA and protein levels (31).

Vitamin E supplementation: While early trials showed mixed results, recent studies using water-soluble formulations with improved bioavailability have renewed interest. The optimal dosing window appears critical, with supplementation most effective when initiated within 48 h of birth (32, 33).

Lutein and zeaxanthin: These macular carotenoids accumulate in the developing retina during the third trimester. Studies have shown reduced oxidative stress markers and trends toward lower ROP incidence, though larger trials are needed (34).

Melatonin: Beyond its antioxidant properties, melatonin modulates VEGF expression and promotes anti-inflammatory pathways. A pilot study of oral melatonin (0.5 mg/kg) showed reduced inflammatory cytokines and a trend toward lower ROP severity (35).

#### 3.2.4 Hypoxia-inducible factor (HIF) stabilization

HIF-1α stabilization represents a paradigm shift from anti-angiogenic to pro-physiological vascularization strategies. During the first phase of ROP, relative hyperoxia destabilizes HIF-1α, halting normal vessel growth. Prolyl hydroxylase domain (PHD) inhibitors stabilize HIF-1α, promoting controlled vascular development (36).

Roxadustat, an oral PHD inhibitor approved for anemia treatment, has shown promise in preclinical ROP models. Low-dose treatment during the hyperoxic phase preserved normal vascular development and reduced subsequent neovascularization (37). However, these findings are from preclinical ROP models, and the translatability of this critical therapeutic window to human infants, as well as the long-term safety of systemic HIF stabilization, remains to be established.

Novel delivery approaches using nanoparticle formulations enable targeted retinal delivery while minimizing systemic effects. Proof-of-concept studies demonstrate that intravitreal PHD inhibitor-loaded nanoparticles promote revascularization of avascular retina without inducing pathological neovascularization (38).

#### 3.2.5 Cell-based therapies

Mesenchymal stem cells (MSCs) offer unique advantages through paracrine effects, immunomodulation, and tissue regeneration capabilities. Multiple preclinical studies demonstrate that intravitreal MSC injection during early ROP reduces vaso-obliteration and subsequent neovascularization (39). While mechanistically compelling, significant hurdles in cell sourcing, standardization, dosing, and long-term safety (e.g., immunogenicity, tumorigenicity) must be addressed before this approach can be considered for human clinical trials.

Human umbilical cord-derived MSCs (hUC-MSCs) show particular promise due to their accessibility, low immunogenicity, and robust secretome. These cells secrete multiple protective factors including IGF-1, VEGF-A165 (physiological isoform), angiopoietin-1, and anti-inflammatory cytokines (40).

Exosomes derived from MSCs represent a cell-free alternative that maintains therapeutic efficacy while potentially reducing safety concerns. MSC-derived exosomes contain microRNAs, proteins, and lipids that modulate angiogenesis, reduce inflammation, and promote tissue repair (41). Preclinical studies show that intravitreal exosome injection achieves similar efficacy to whole-cell therapy with improved safety profile.

#### 3.2.6 Beta-adrenergic receptor blockade

The adrenergic system has emerged as a crucial regulator of retinal angiogenesis. Preclinical studies using the oxygen-induced retinopathy (OIR) model have demonstrated that retinal hypoxia triggers a surge in norepinephrine release, which stimulates β2-and β3 adrenoreceptors (ARs). This stimulation subsequently upregulates pro-angiogenic factors, including VEGF and HIF-1α, driving pathological neovascularization (42, 43). Crucially, blockade of these receptors selectively inhibits pathological neovascularization without interfering with physiological retinal vascular development, offering a distinct safety advantage over direct VEGF inhibition (42).

Clinical translation of these findings has focused on propranolol, a non-selective beta-blocker. Several randomized controlled trials (RCTs) and prospective studies investigating oral propranolol (typically 1–2 mg/kg/day) have reported a reduction in ROP progression to advanced stages and a decreased need for rescue therapies (laser or anti-VEGF) (44). For instance, a multicenter RCT by Filippi et al. showed reduced progression to stage 3 ROP (45). Recent meta-analyses confirm that prophylactic oral propranolol significantly reduces the risk of severe ROP and the need for invasive treatment (46).

However, systemic safety remains a primary concern. Oral propranolol has been associated with adverse events such as bradycardia, hypotension, and apnea, particularly in unstable preterm infants with comorbidities (45). To mitigate these systemic risks, recent research has pivoted toward topical administration. While 0.1% drops were found to be well-tolerated but insufficiently effective, dose-optimization studies continue to refine this approach (47).A phase II trial of 0.2% propranolol eye micro-drops demonstrated a reduction in ROP progression with significantly lower plasma drug levels compared to oral administration, suggesting a safer therapeutic window (48) Table 1.

**Table 1:** Summary of emerging therapeutic targets for ROP (beyond anti-VEGF).

Therapeutic category	Mechanism of action	Key evidence	Evidence strength	Clinical status
IGF-1/IGF-1/IGFBP3 Complex	Promotes hysiological vascularization; deficiency is pathogenic	NIRTURE Phase II trial showed promising trends (though not powered for ROP)	Moderate (Human)	Phase II/III clinical trials
Omega-3 PUFAs (DHA/EPA)	Anti-inflammatory (via resolvins) and anti-angiogenic properties	Systematic review suggests fish oil emulsions may reduce severe ROP. OIR models.	Moderate (Human/Preclinical)	Clinical (Lipid emulsion formulations)/Preclinical (SPMs)
Antioxidant Therapies	Target oxidative stress pathways central to ROP pathogenesis	NAC: Preclinical models show reduced neovascularization. Melatonin: Pilot study showed reduced inflammatory markers	Low-to-Moderate	Preclinical/Pilot Studies
HIF stabilization (e.g., roxadustat)	Stabilizes HIF-1*α* to promote physiological vascularization in Phase I ROP	Preclinical OIR models showed preserved vascular development	Low (Preclinical)	Preclinical
Cell-Based Therapies (MSCs, Exosomes)	Paracrine effects, immunomodulation, and secretion of protective factors (e.g., IGF-1)	Multiple preclinical studies show reduced neovascularization. Exosome studies are also preclinical	Low (Preclinical)	Preclinical
Beta-Blockers (Propranolol)	Blocks hypoxia-induced norepinephrine stimulation of β-ARs, downregulating VEGF/HIF-1α; spares physiological vessels	Meta-analyses show reduced progression to severe ROP and reduced need for laser/anti-VEGF	High (RCTs & Meta-analyses)	Oral: Clinical use (off-label); Topical: Phase II Trials

### 3.3 Novel biomarkers for ROP

#### 3.3.1 Serum biomarkers

##### 3.3.1.1 IGF-1 and IGFBP-3

Serum IGF-1 levels have emerged as one of the most validated biomarkers for ROP risk stratification. The WINROP (Weight, IGF-1, Neonatal ROP) algorithm combines longitudinal IGF-1 measurements with weight gain patterns to predict ROP development. Validation studies across multiple populations demonstrate sensitivity of 100% for detecting severe ROP, with the ability to reduce screening burden by 84% (49).

Recent refinements include the incorporation of IGFBP-3 levels, which stabilize IGF-1 and provide additional predictive value. The IGF-1/IGFBP-3 molar ratio at 34 weeks postmenstrual age shows stronger correlation with ROP severity than either biomarker alone (50). Machine learning algorithms integrating these biomarkers with clinical parameters achieve area under the curve (AUC) values exceeding 0.95 for predicting treatment-requiring ROP (51).

##### 3.3.1.2 Inflammatory markers

Systemic inflammation plays a crucial role in ROP pathogenesis. A comprehensive proteomic analysis identified a panel of inflammatory biomarkers with predictive value:

• C-reactive protein (CRP): Elevated CRP levels during the three weeks of life correlate with ROP severity. Serial measurements showing persistent elevation predict progression to severe ROP (52).

Inflammatory cytokine spectrum: The dynamic changes in cytokine profiles offer predictive value for ROP.Multiple cytokines exhibit temporal trends: In preterm infants, elevated IL-6 levels and decreased IL-17/IL-18 levels at birth, or increased CRP and reduced NT-4 levels on day 3 postnatal; or elevated IL-18 with decreased TGF-β, BDNF, and RANTES levels during the first 1–3 weeks after birth. These patterns collectively indicate potential ROP development (52).

Soluble E-selectin (sE-selectin): This endothelial activation marker demonstrates superior performance to traditional inflammatory markers. Levels >43 ng/mL predict ROP with 83% sensitivity and 60% specificity (53).

#### 3.3.2 Metabolomic profiling

Untargeted metabolomics has revealed novel biomarkers reflecting altered metabolism in ROP:

Amino acid signatures: Infants with severe retinopathy of the developing retina (ROP) present a specific metabolic pattern characterized by elevated levels of arginine, glutamate, citrulline, and tryptophan, and decreased levels of lysine and aspartate. Studies have found that the degree of elevated arginine and glutamine levels can predict the severity of ROP (54).

Lipid mediators: Lipidomic analysis identifies reduced levels of anti-inflammatory lipid mediators (resolvins, protectins) and elevated pro-inflammatory eicosanoids in infants developing ROP (55).

One-carbon metabolism: Altered folate and methionine metabolism, reflected by elevated homocysteine and reduced S-adenosylmethionine levels, correlates with ROP severity. These changes may reflect epigenetic dysregulation and oxidative stress (56).

#### 3.3.3 MicroRNA signatures

Circulating microRNAs offer advantages as biomarkers due to their stability and tissue-specific expression patterns. Recent studies have identified several promising candidates:

miR-200b: This microRNA regulates angiogenesis through VEGF modulation. Serum levels show inverse correlation with ROP severity. Low miR-200b predicts progression to severe ROP (57).

miR-221: Over-expression of miR-221 inhibited the growth of endothelial cells *in vitro* by targeting the c-kit receptor. A study in zebra fish showed that miR-221 deficiency resulted in drastic developmental vascular defects which underscore an important function of miR-221 in angiogenesis. A rapid decline in miR-221 levels indicates increased ROP risk (58).

miR-126: This endothelial-enriched microRNA regulates vascular development. Combined measurement of miR-126 and its complementary strand miR-126* improves predictive accuracy, with the miR-126/miR-126* ratio showing strongest correlation with ROP outcomes (59).

MicroRNA panels: Machine learning approaches combining multiple microRNAs achieve superior performance. A seven-microRNA signature (miR-106a, miR-146, miR-181, miR-199a, miR-214, miR-424, miR-451) predicts treatment-requiring ROP (60).

#### 3.3.4 Advanced imaging biomarkers

##### 3.3.4.1 OCT and OCT-angiography

Optical coherence tomography (OCT) provides high-resolution imaging of retinal microstructure, revealing subclinical changes preceding visible ROP:

Foveal development: Delayed foveal maturation, evidenced by persistent inner retinal layers and shallow foveal depression, predicts ROP progression. Quantitative metrics including median central foveal thickness, median inner retinal layer thickness, and the median foveal-to-parafoveal thickness ratio at 31–36 weeks show strong correlation with final ROP severity (61).

Choroidal changes: Subfoveal choroidal thickness measurements reveal early vascular compromise. Progressive choroidal thinning indicates high risk for severe ROP development (62).

OCT-angiography: This non-invasive technique quantifies retinal vascular density and complexity. Reduced parafoveal vessel density and increased vascular tortuosity precede clinical ROP, offering opportunity for earlier intervention (63).

##### 3.3.4.2 Artificial intelligence-enhanced screening

Deep learning algorithms analyzing retinal images have revolutionized ROP screening:

Automated severity grading: Convolutional neural networks achieve expert-level performance in detecting plus disease and stage classification. The i-ROP DL system demonstrates 93% sensitivity and 94% specificity for identifying treatment-requiring ROP (64).

Predictive modeling: AI systems integrating imaging features with clinical data predict ROP progression more accurately than either modality alone. Temporal analysis of vascular changes enables prediction of treatment need before conventional criteria (65).

Quantitative vascular analysis: Automated measurement of vascular tortuosity, diameter, and branching patterns provides objective metrics. The tortuosity index at posterior pole combined with peripheral vascular density was 95% accurate in predicting ROP (66).

#### 3.3.5 Challenges in biomarker validation and implementation

While the biomarkers cataloged above show potential, their transition from research to routine clinical use faces significant hurdles. Many findings originate from single-center or small-scale pilot studies and lack large-scale, multi-center validation in diverse patient populations. Standardization of assays is a major challenge; for example, metabolomic and microRNA profiles can be highly sensitive to sample collection, processing, and analysis techniques, leading to poor reproducibility across different laboratories.

Furthermore, clinical feasibility and cost-effectiveness must be considered. Advanced imaging like OCT-A and deep learning systems require substantial capital investment and specialized training, limiting their accessibility in lower-resource settings. Serum-based algorithms like WINROP are more scalable, but the cost and rapid turnaround time required for serial IGF-1 measurements must be proven practical for routine NICU workflows. Future research must focus not only on discovery but also on rigorous validation, standardization, and health-economic analyses to prove clinical utility.

### 3.4 Combination therapy approaches

#### 3.4.1 Rationale for combination treatment

The multifactorial pathogenesis of ROP suggests that targeting multiple pathways simultaneously may enhance efficacy while reducing individual drug doses and associated toxicity. Combination approaches aim to address both phases of ROP: preventing vaso-obliteration during phase I while controlling neovascularization in phase II (67).

#### 3.4.2 Anti-VEGF plus Omega-3 supplementation

Combining anti-VEGF therapy with omega-3 fatty acids leverages complementary anti-inflammatory and anti-angiogenic mechanisms:

The PROP-ROP trial is investigating whether post-treatment omega-3 supplementation reduces ROP reactivation after anti-VEGF therapy. Preliminary results suggest 60% reduction in reactivation rates with combined treatment (68).

Mechanistically, omega-3-derived specialized pro-resolving mediators enhance resolution of inflammation following anti-VEGF treatment, promoting healing and reducing scarring. Resolvin E1 administration following anti-VEGF injection accelerates retinal vascular maturation in animal models (69).

#### 3.4.3 Antioxidant cocktails

Recognizing that oxidative stress involves multiple pathways, combination antioxidant approaches show promise:

Coenzyme Q10 plus vitamin E: Current research found that the use of Q10/Vitamin E combination coenzyme provided faster retinal maturation with fewer treatments in infants with ROP. This appears compatible with the biological effects of the CQ10 and Vitamin E complex which is shown to support antioxidant defense systems and suppress toxic effects of the free oxygen radicals in premature infants and thereby reduce the incidence of ROP (70).

Lutein and zeaxanthin: Studies have reported that lutein and zeaxanthin supplementation during pregnancy can effectively reduce the incidence of retinopathy in preterm infants, mainly by reducing pathological hypoxia stimulation of vascular endothelial cells to control vascular loss in hypoxic stress environment, so as to protect and improve oxygen-induced retinopathy (71).

#### 3.4.4 Sequential therapy protocols

Time-sensitive interventions targeting different disease phases represent an emerging strategy, For instance, two reviews suggested that sequential treatment regimens may help reduce the occurrence of severe ROP compared with standard treatment regimens (72, 73):

Phase I intervention (birth to 32 weeks PMA): using exogenous EPO, preventing hyperglycemia and supplementing with IGF-1, omega-3 fatty acids to promote physiological vascularization and reduce inflammation (72).

Transition phase (32–34 weeks PMA): Avoiding exogenous EPO in this phase may prevent further increase in retinal EPO. Omega-3 supplements suppress TNF-α and vitamin E suppresses oxidative stress (72).

Phase II intervention (>34 weeks PMA): In addition to targeting oxygen saturation, correcting anemia with PRBC transfusion and exposing infants to light may improve retinal hypoxia and decrease VEGF. Low-dose anti-VEGF if proliferative disease develops, with continuation of supportive therapies (72).

### 3.5 Practical implementation considerations for clinicians

The translation of novel therapeutic approaches into clinical practice requires careful consideration of several practical aspects:

**Risk-benefit assessment:** When considering alternative therapies, clinicians must weigh potential benefits against the established the immediate sight-saving benefits of anti-VEGF therapy may outweigh theoretical lo efficacy of conventional treatments. For infants with zone I or aggressive posterior ROP, ng-term concerns (74). Conversely, for less severe disease, approaches with better safety profiles may be preferable even if efficacy is comparable rather than superior.

**Integration with existing protocols:** Novel treatments should be integrated into established ROP screening and treatment protocols. For example, biomarker testing could be scheduled alongside routine blood sampling to minimize procedural burden, while IGF-1 supplementation might be incorporated into nutritional management protocols (75).

**Resource stratification:** Implementation strategies should be tailored to resource availability. High-resource settings might implement comprehensive approaches combining advanced biomarker testing with personalized treatment protocols, while resource-limited settings could prioritize cost-effective interventions like nutritional optimization and affordable antioxidant supplementation (76).

**Multidisciplinary collaboration:** Effective ROP management requires coordination between neonatologists, ophthalmologists, nutritionists, and pharmacy services. Establishing clear communication pathways and standardized protocols is essential, particularly for implementing combination therapy approaches (77).

### 3.6 Clinical translation and implementation challenges

#### 3.6.1 Regulatory considerations

The translation of novel ROP therapies faces unique regulatory challenges:

Pediatric drug development: The small market size and ethical considerations of clinical trials in premature infants create barriers. The FDA's Pediatric Drug Development Program provides incentives, but ROP-specific guidance remains limited (78).

Combination therapy approval: Regulatory pathways for combination therapies are complex, particularly when combining approved drugs (anti-VEGF) with investigational agents. Adaptive trial designs may facilitate development (79).

Biomarker validation: Regulatory acceptance of biomarkers for patient selection or treatment monitoring requires extensive validation across diverse populations. The FDA's Biomarker Qualification Program provides a framework but requires substantial evidence (80).

#### 3.6.2 Economic considerations

Cost-effectiveness analyses reveal important insights:

Biomarker screening: Implementation of WINROP or similar algorithms could reduce screening costs while maintaining safety. However, initial setup costs and training requirements present barriers (81).

Combination therapies: While potentially more expensive initially, reduced retreatment rates and improved outcomes may offer long-term cost savings. Economic modeling suggests break-even within 3 years when considering reduced visual disability (82).

#### 3.6.3 Implementation barriers

Several challenges impede clinical adoption:

Infrastructure requirements: Advanced biomarker testing and specialized drug preparation may not be available in all neonatal units. Centralized testing models and point-of-care devices are under development (83).

Training needs: Implementation of new therapies requires extensive staff education. Telemedicine models for expert consultation show promise in addressing expertise gaps (84).

Global health disparities: Novel therapies may widen the gap between high and low-resource settings. Simplified protocols and affordable alternatives are crucial for global impact (85).

## 4 Future directions

### 4.1 Emerging technologies

Several cutting-edge approaches show promise for future ROP management:

Gene therapy: AAV-mediated delivery of protective factors (IGF-1, PEDF) offers potential for sustained therapeutic effect with single administration. Preclinical studies demonstrate long-term efficacy and safety (86).

Nanotechnology: Targeted nanoparticle delivery systems enable precise drug delivery to the retina while minimizing systemic exposure. Biodegradable nanoparticles loaded with multiple therapeutic agents achieve sustained release over weeks (87).

Tissue engineering: Bioengineered retinal vessels using induced pluripotent stem cells may eventually enable replacement of damaged vasculature. Proof-of-concept studies show integration of engineered vessels with host retina (88).

### 4.2 Personalized medicine approaches

The integration of multi-omic data promises truly personalized ROP management:

Risk stratification algorithms: Machine learning models integrating genomic, proteomic, metabolomic, and clinical data achieve unprecedented predictive accuracy. Cloud-based platforms enable real-time risk assessment.

Therapeutic selection: Pharmacogenomic markers may guide treatment selection. Variants in VEGF pathway genes influence anti-VEGF response, while IGF1 polymorphisms affect supplementation efficacy (89).

Outcome prediction: Long-term visual and neurodevelopmental outcomes can be predicted using early biomarkers, enabling targeted early intervention services.

## 5 Conclusions

The landscape of ROP management is rapidly evolving beyond exclusive reliance on anti-VEGF therapy. Novel therapeutic targets addressing fundamental pathogenic mechanisms show potential for more effective and safer treatments. IGF-1 supplementation, omega-3 fatty acids, antioxidants, HIF stabilizers, and cell-based therapies each show promise. However, significant evidence gaps must be addressed. The safety and efficacy of most non-VEGF therapies have not yet been confirmed in large-scale, Phase III randomized controlled trials. Combination approaches, while mechanistically appealing, require rigorous investigation to define optimal dosing and rule out antagonistic effects.

The development of robust biomarkers is crucial for enabling risk stratification and personalized treatment selection. From simple serum markers to sophisticated imaging analyses and multi-omic signatures, these tools have the potential to transform ROP screening and management. Crucially, most novel biomarkers require multi-center validation to confirm their predictive value, reproducibility, and clinical utility before they can be widely adopted.

Successful clinical translation requires addressing regulatory, economic, and implementation challenges. The ultimate goal—preventing blindness while optimizing overall outcomes for premature infants—may become more achievable through these innovative approaches. The next decade could witness a paradigm shift in ROP management, moving from reactive treatment of established disease to proactive, personalized interventions that promote healthy retinal development from birth. This transformation promises to significantly reduce the global burden of ROP-related visual impairment while serving as a model for precision medicine in neonatal care.
